# Through network pharmacology and molecular docking to explore the underlying mechanism of *Artemisia annua* L. treating in abdominal aortic aneurysm

**DOI:** 10.3389/fphys.2022.1034014

**Published:** 2022-10-20

**Authors:** Longyuan Jia, Yuchen Jing, Ding Wang, Shuai Cheng, Chen Fu, Xiangyu Chu, Chenye Yang, Bo Jiang, Shijie Xin

**Affiliations:** ^1^ Department of Vascular Surgery, The First Affiliated Hospital of China Medical University, Shenyang, China; ^2^ Key Laboratory of Pathogenesis, Prevention, and Therapeutics of Aortic Aneurysm in Liaoning Province, Shenyang, China; ^3^ Department of Pharmacology, School of Pharmacy, China Medical University, Shenyang, Liaoning, China

**Keywords:** abdominal aortic aneurysm, *Artemisia annua* L., molecular docking, network pharmacology, therapeutic targets

## Abstract

**Background:** Abdominal aortic aneurysm (AAA) is a degenerative disease that causes health problems in humans. However, there are no effective drugs for the treatment of AAA. *Artemisia annua* L. (*A. annua*) is a traditional herbal that has been widely used in cardiovascular disease. Based on network pharmacology and molecular docking technology, this study predicted the practical components and potential mechanisms of *A. annua* inhibiting the occurrence and development of AAA.

**Methods:** The main active ingredients and targets of *A. annua* were screened through the TCMSP database; the GeneCards, OMIM, PharmGkb, and TTD databases were used to search for the targeted genes of AAA and map them to the targets of the active ingredients to obtain the active ingredient therapy of *A. annua*. The targets of AAA were to construct a protein interaction network through the STRING platform. R software was used to carry out the enrichment analysis of GO and KEGG for relevant targets, and Cytoscape was used to construct the active ingredient-target network prediction model of *A. annua*. Finally, AutoDock Vina was used to verify the results of the active ingredients and critical targets.

**Results:** The main active ingredients obtained from *A. annua* for the treatment of AAA include quercetin, luteolin, kaempferol, isorhamnetin, and artemetin, as well as 117 effective targets, including RELA, MAPK14, CCND1, MAPK1, AKT1, MYC, MAPK8, TP53, ESR1, FOS, and JUN. The 11 targeted genes might play a key role in disease treatment. Enriched in 2115 GO biological processes, 159 molecular functions, 56 cellular components, and 156 KEGG pathways, inferred that its mechanism of action might be related to PI3K-Akt signaling pathway, fluid shear stress, atherosclerosis, and AGE-RAGE signaling pathway. Molecular docking results showed that the top five active components of *A. annua* had a good affinity for core disease targets and played a central role in treating AAA. The low binding energy molecular docking results provided valuable information for the development of drugs to treat AAA.

**Conclusion:** Therefore, *A. annua* may have multiple components, multiple targets, and multiple signaling pathways to play a role in treating AAA. *A. annua* may have the potential to treat AAA.

## Introduction

Abdominal aortic aneurysm (AAA) is mainly characterized by local progressive dilation of the abdominal aorta, the most high-risk vascular degenerative disease in vascular surgery (Kugo et al., 2019). Once AAA ruptures, the mortality rate can reach 80%. After surgical resuscitation, mortality remains high at around 42% (Karthikesalingam et al., 2014). AAA is usually diagnosed when the diameter of the upper abdominal aorta is greater than 30 mm (Moll et al., 2011). Currently, AAA with greater than 55 mm in diameter is mainly treated by surgical intervention, and these surgical interventions are effective ways to prevent abdominal aortic rupture (Powell, 1998; Lavin et al., 2019). In addition to surgical treatment, there is currently a lack of effective drug interventions, especially in the early treatment of AAA (Baxter et al., 2008; Golledge et al., 2017). Therefore, it is vital to explore potential effective drugs.

Chinese traditional medicine is a substantial medical resource. *Artemisia annua* L. (*A. annua*) is a kind of traditional Chinese medicine. With the award of the 2015 Nobel Prize in Physiology or Medicine to a Chinese scientist, Artemisia has attracted global attention (Abba et al., 2018). *Artemisia* and its derivatives are extensively used to treat oncology and cardiovascular diseases (Bora and Sharma, 2011; von Hagens et al., 2017; Abba et al., 2018; Saeed et al., 2019; Aktaş et al., 2020). Several studies have shown that *A. annua* and its derivatives have a particular therapeutic effect on inhibiting atherosclerosis and inflammation (Cao et al., 2020; He et al., 2020; Jiang et al., 2020). Although *A. annua* contains a variety of active ingredients, its therapeutic target and mechanism for AAA treatment are not fully understood.

**FIGURE 1:**
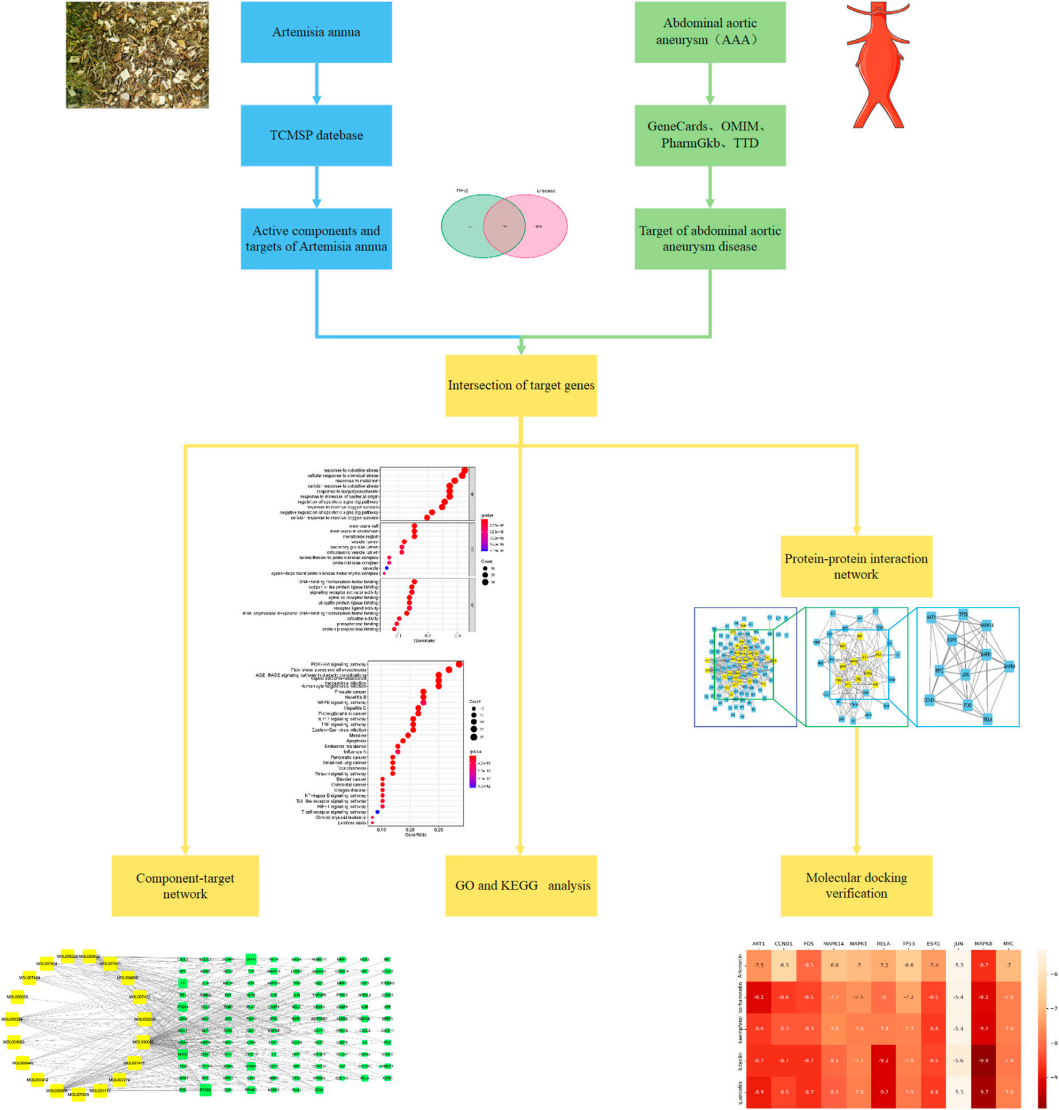
The flowchart of this study.

Network pharmacology, based on bioinformatics and computer technology, integrates a large amount of biological information and data to study the mechanism of action of multi-target drugs from molecules to cells to the body (Hopkins, 2008; Berger and Iyengar, 2009). The strength of network pharmacology lies in analyzing the “drug-component-target-disease” interaction network, systematically discovering drug-disease associations, and revealing the synergistic effects between multi-molecular drugs (Li et al., 2014). Furthermore, molecular docking is a statistical simulation method that focuses on the interaction between molecules and predicts their binding mode and affinity (Wang and Zhu, 2016). The main function of the method is to identify the binding pocket and binding affinity of the drug to the target protein. Therefore, with the help of network pharmacology and molecular docking methods, this study analyzed the role and mechanism of *A. annua* in the treatment of AAA, aiming to provide new ideas for drug treatment of AAA and to facilitate new drug development in the future. The flowchart for this study was shown in Figure 1.

## Materials and methods

### Database and software

①Drug component target database: Traditional Chinese Medicine Systems Pharmacology Database and Analysis Platform (TCMSP, http://tcmspw.com/tcmsp.php). ②Disease Target Database: GeneCards (https://www.genecards.org/); Online Mendelian Inheritance in Man (OMIM, https://omim.org), Pharmacogenomics Knowledgebase (PharmGKB, https://www.pharmgkb.org/); Therapeutic Target Database (TTD, http://db.idrblab.net/ttd/); ③Protein database, UniProt (https://www.uniprot.org); Protein Data Bank (PDB, http://www.rcsb.org/); ④Protein interaction analysis platform, String (https://String-db.org/); ⑤Network analysis and mapping software: Cytoscape 3.8.0;R (R4.0.3 for Windows); ⑥Biological information analysis packet: VennDiagram packet; Bioconductor(https://Bioconduct.org/biolite.r) and its: org.hs.eg.DB, ⑦Molecular Docking Software: AutoDock Vina 4.1, PyMOL 2.4.

### Collection of the active components and targets of *A. annua*

All active ingredients in *A. annua* were obtained from TCMSP (https://www.tcmspw.com/tcmsp.PHP). The classification standards were defined based on drug-likeness (DL) greater than or equal to 0.18 and oral bioavailability (OB) greater than or equal to 30% (Xu et al., 2020). Then, the targets of the selected compounds were obtained from the TCMSP database, and the targeted name was input into Uniprot (http://www.uniprot.org/) to obtain the standardized gene symbol.

### Screening of genes related to the treatment of AAA with *A. annua*

In the Genecards, OMIM, PHARGKB, and TDD databases, “abdominal aortic aneurysm” was input as the keyword for retrieval to obtain related AAA targets. The Venn diagram packet was then run in R to obtain compositional targets of *A. annua* intersected with targets related to AAA to screen out the targets related to the treatment of AAA in *A. annua.*

### GO and KEGG pathway enrichment analysis

The ClusterProfiler software package in R software (version 4.0.3) was used for Gene Ontology (GO) and Kyoto Encyclopedia of Genes and Genomes (KEGG) pathway enrichment analysis of the intersection genes (Kanehisa and Goto, 2000; Yu et al., 2012). When the q value ≤0.05, GO terms and KEGG pathways were considered to be statistically significant. Then, the top 10 GO terms and the top 30 KEGG pathways for molecular function (MF), cellular component (CC), and biological process (BP) were selected for further analysis.

### Construction of the component-target network

The targets of *A. annua* for treating AAA were input into Cytoscape software to construct a “component-target” network (Shannon, 2003). The active components and targets of the drug were represented as “nodes”, and the interaction between nodes was defined as “edges".

### PPI network construction and core target screening

The intersection gene data were imported into the String database (https://string-db.org/) to obtain the possible intersection points and establish the relationship between the targets (Szklarczyk et al., 2019). The generated files were then imported into Cytoscape software for protein-protein interaction (PPI) maps to describe the relationship between *A. annua* and the intersecting genes of AAA.

### Molecular docking

Referring to the previous research literature (Powell, 1998; Hong et al., 2022; Xu et al., 2022), we selected the top 5 active ingredients in *A. annua* as ligands and hub genes from the PPI network as receptors for molecular docking validation. According to the molecular docking method, the 3D structure of the active component was downloaded from PubChem CID, the protein structure guide of the target was downloaded from the PDB database, and the hydrodewatering and hydrogenation of the protein were carried out by using PyMol software (Seeliger and de Groot, 2010; Lill and Danielson, 2011). The component and target protein formats were entered into PDBQT format by AutoDockTools1.5 (Morris et al., 2008). Molecular docking was performed using AutoDock Vina 4.1 software, and the results - were further analyzed by PyMol 2.4 (Trott and Olson, 2010).

## Results

### Screening of active ingredients and targets of *A. annua* for the treatment of AAA

After the search, screening was carried out under the conditions of OB greater than or equal to 30% and DL greater than or equal to 0.18, and the nontarget components were removed. The 22 potential effective components were obtained (Table 1). Furthermore, we found that 510 potential targets corresponded to 22 potential effective ingredients (Supplementary Table S1).

**TABLE 1:** The main active ingredients of *A. annua*.

MOL ID	Molecule name	OB	Dl
MOL002235	Eupatin	50.8	0.41
MOL000354	Isorhamnetin	49.6	0.31
MOL000359	Sitosterol	36.91	0.75
MOL004083	Tamarixetin	32.86	0.31
MOL004112	Patuletin	53.11	0.34
MOL000422	Kaempferol	41.88	0.24
MOL000449	Stigmasterol	43.83	0.76
MOL004609	Areapillin	48.96	0.41
MOL005229	Artemetin	49.55	0.48
MOL000006	Luteolin	36.16	0.25
MOL007274	Skrofulein	30.35	0.3
MOL007389	Artemisitene	54.36	0.31
MOL007400	Vicenin-2_qt	45.84	0.21
MOL007401	Cirsiliol	43.46	0.34
MOL007404	Vitexin_qt	52.18	0.21
MOL007412	DMQT	42.6	0.37
MOL007415	[(2S)-2-[[(2S)-2-(benzoylamino)-3-phenylpropanoyl]amino]-3-phenylpropyl] acetate	58.02	0.52
MOL007423	6,8-di-c-glucosylapigenin_qt	59.85	0.21
MOL007424	Artemisinin	49.88	0.31
MOL007425	Dihydroartemisinin	50.75	0.3
MOL007426	Deoxyartemisinin	54.47	0.26
MOL000098	Quercetin	46.43	0.28

### Targets of *A. annua* for AAA

**FIGURE 2:**
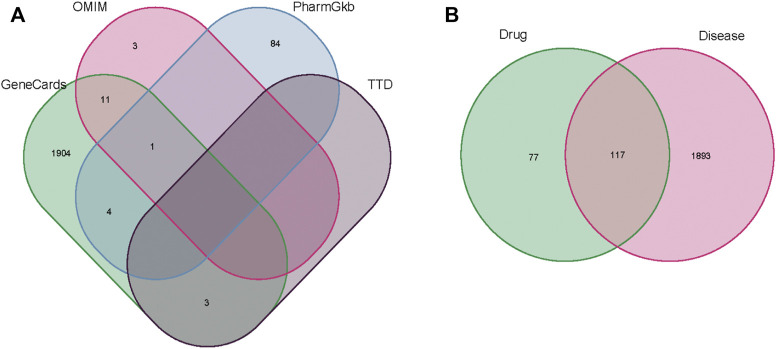
**(A)** The results of the Venn diagram of AAA-related targets in four databases. **(B)** The results of the Venn diagram of drug genes (green) and disease genes (pink).

The related targets of AAA were collected from the GeneCards, OMIM, PharmGkb, and TTD databases. The data were sorted and merged to obtain a total of 2010 disease targets (Supplementary Table S2), as shown in Figure 2A. Then, we intersected the obtained *A. annua* targets with the genes associated with AAA and obtained a Venn diagram of the intersected gene symbols for a total of 117 targets (Supplementary Table S3), as shown in Figure 2B.

### GO enrichment analysis

**FIGURE 3:**
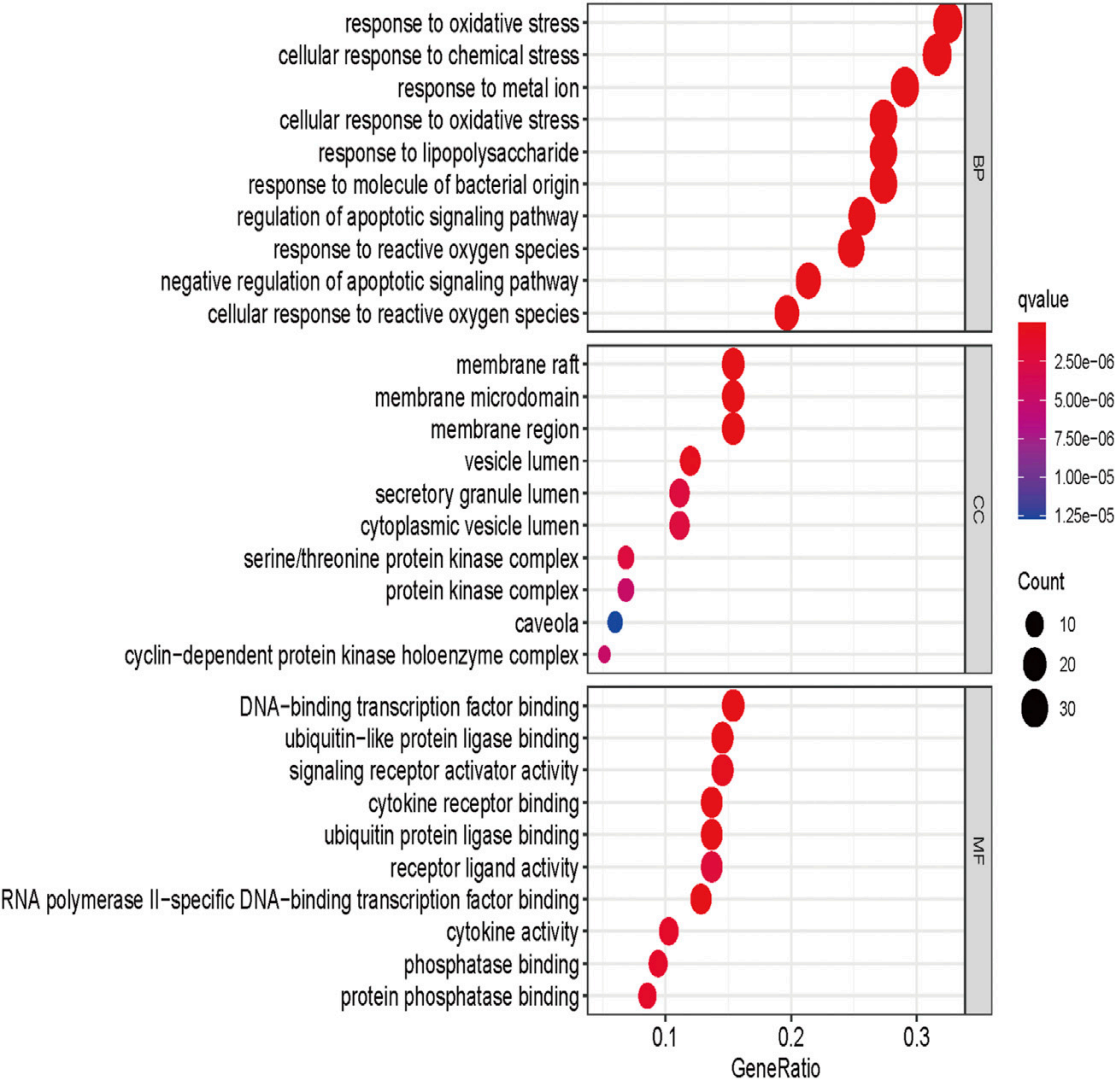
GO enrichment analysis of A. annua compound and AAA “intersection target.”

GO enrichment analysis was performed to analyze 117 genes of drug-disease intersection by using the ClusterProfiler package in R software (version 4.0.3). They grouped the functions of the genes into three components: biological processes (BP), cellular component (CC), and molecular function (MF), and enriched 2115 GO BPs, 159 MFs, and 56 CCs (Supplementary Table S4). The top 10 significant items (*p*-value ≤ 0.05) for each module were shown in Figure 3. The horizontal coordinate indicated the proportion of GO entries, the vertical coordinate representing the name of the enriched entry, and the size of the scatter points represented the number of targets involved in each entry. The higher the significance of the entry, the redder it was. As shown in Figure 3, in biological processes, *A. annua* was mainly associated with oxidative stress, cellular response to chemical stress, and response to metal ion. Among the cellular components, *A. annua* was primarily associated with membrane raft, membrane microdomain, membrane region, and other cellular components. *A. annua* was mainly related to DNA-binding transcription factor binding, ubiquitin-like protein ligase binding, signaling receptor activator activity, and other molecular functions among the molecular functions.

### KEGG enrichment analysis

**FIGURE 4:**
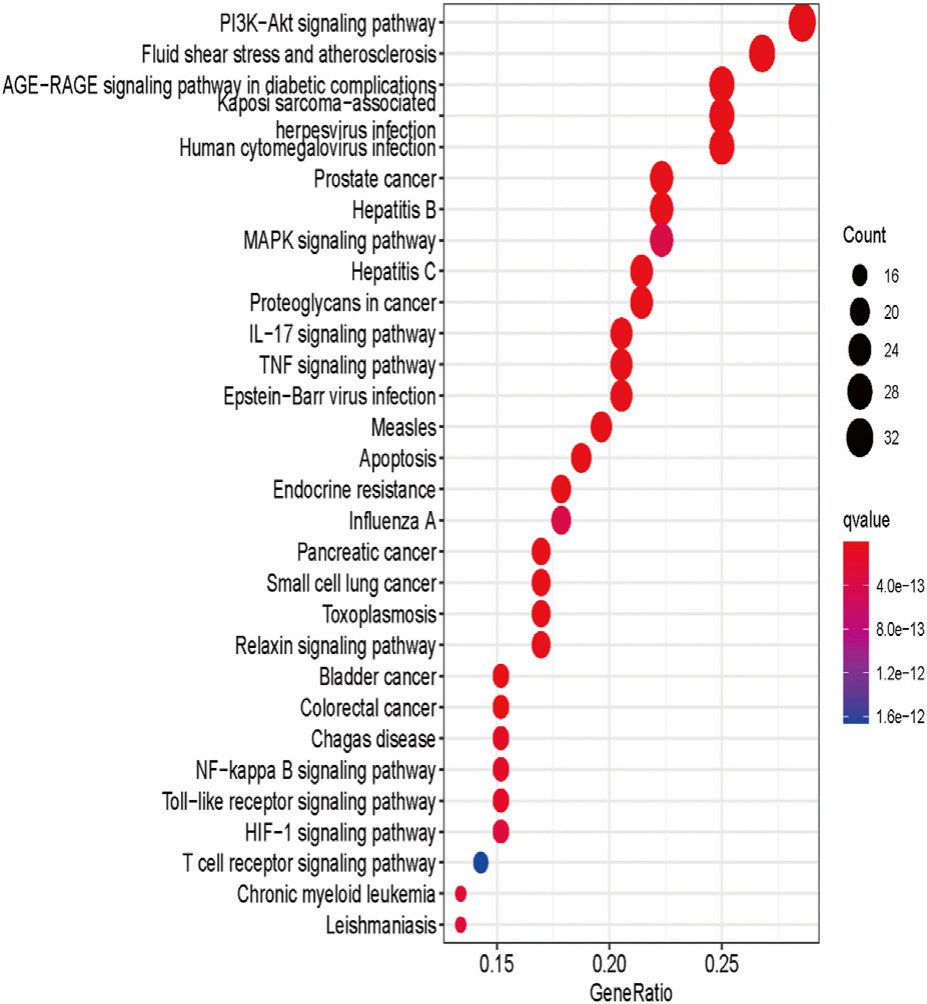
KEGG enrichment analysis of A. annua compound and AAA “intersection target.”

KEGG enrichment analysis was performed using the ClusterProfiler package (version 4.0.3) in R software for 117 genes targeted at drug-disease crossover. The results of KEGG analysis showed that these genes mainly enriched in 156 KEGG pathways (Supplementary Table S5), and the top 30 items were presented in Figure 4. The results suggested that the active ingredients in *A. annua* might act together through multiple pathways, such as the PI3K-Akt signaling pathway, fluid shear stress, atherosclerosis, and AGE-RAGE signaling pathway in diabetic complications. The size and color of the nodes in the bubble map were determined by the number and *p*-value of the associated genes. The node size indicated how many target genes were associated, and the color from purple to yellow reflected the *p*-value from high to low.

### Construction of the *A. annua* component-target network

**FIGURE 5:**
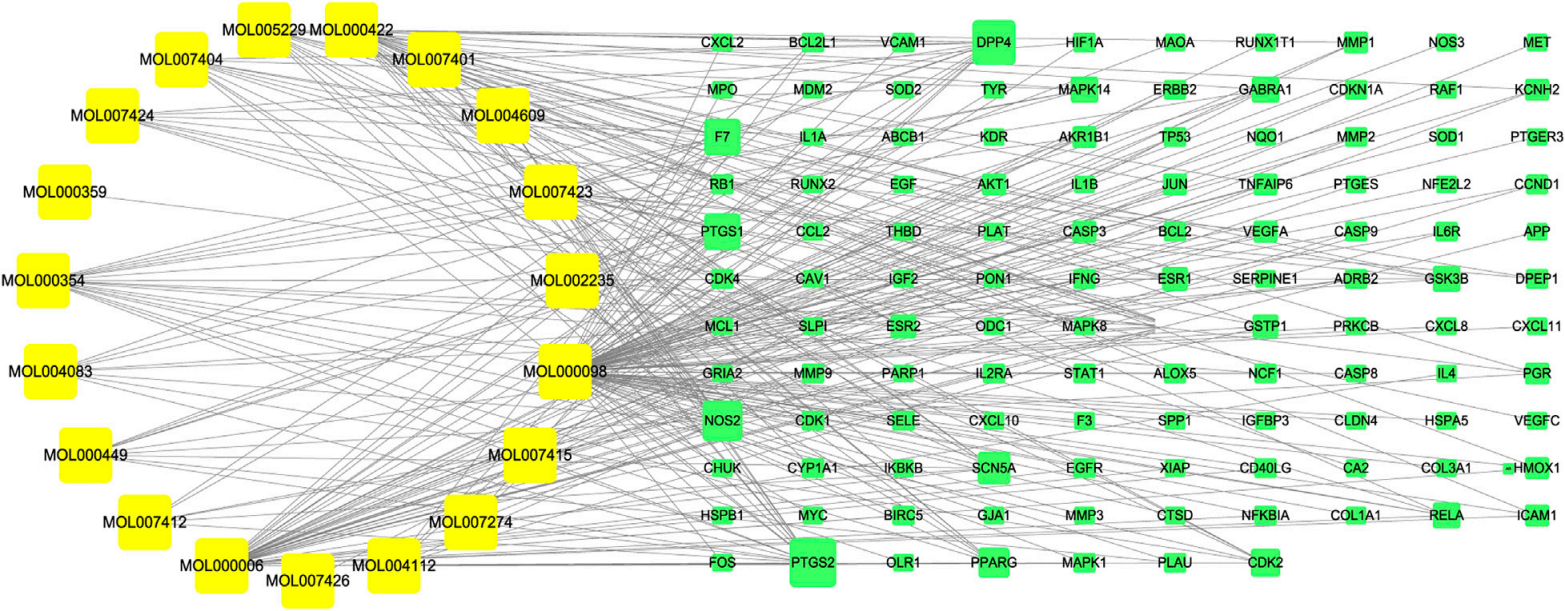
“Component-target” network diagram. Yellow represented the active components of A. annua in the treatment of AAA, and green represented the potential targets.

A total of 117 targets of *A. annua* for treating AAA were input into Cytoscape software to construct a “component-target” network. The active ingredients and targets were represented as “nodes”, and the interaction between nodes was defined as “edges”. The details were presented in Supplementary Table S6. As shown in Figure 5, yellow represented the active components of *A. annua* in the treatment of AAA, and green represented the potential targets.

### Construction of the protein-protein interaction network

**FIGURE 6:**
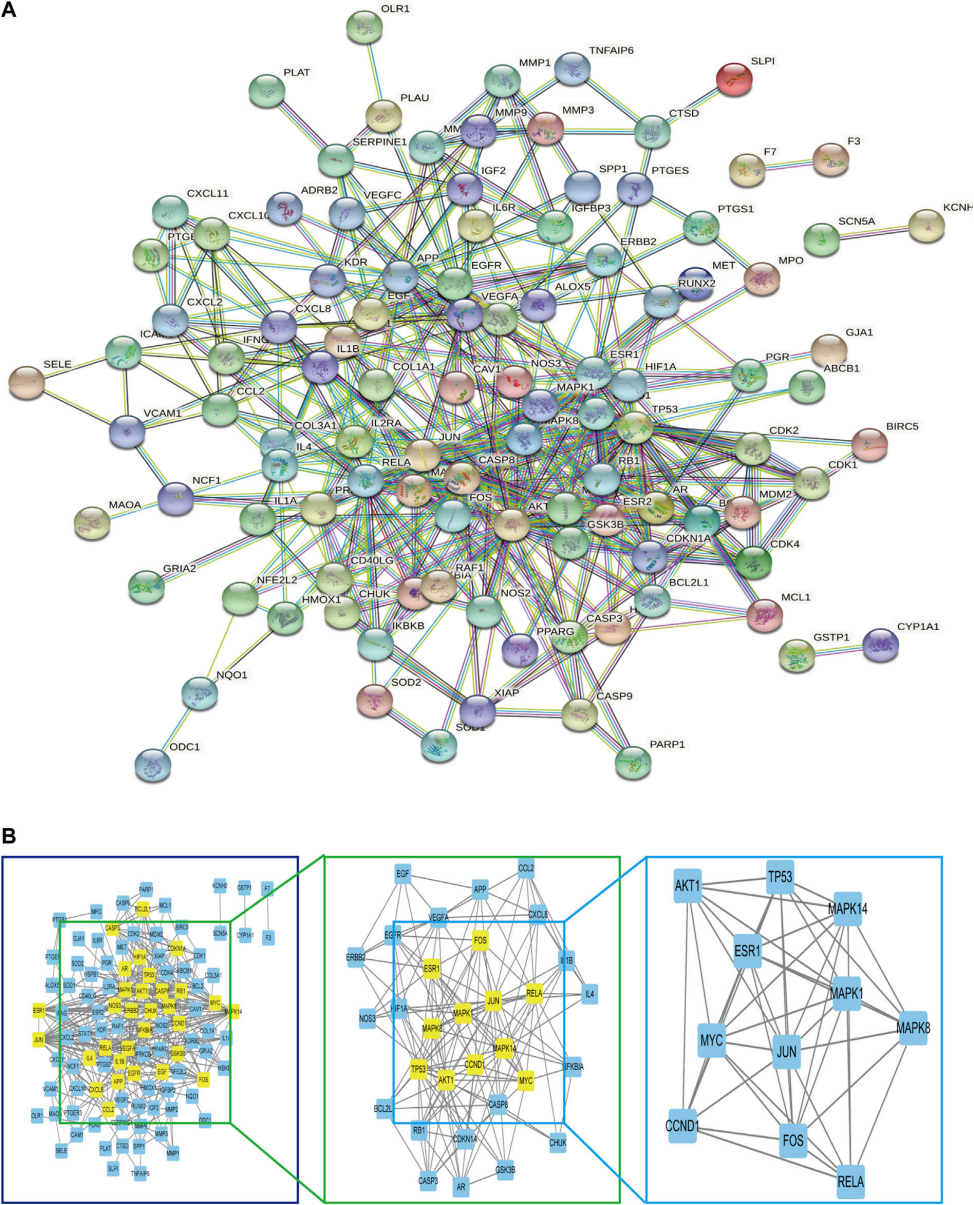
**(A)** Construction of the PPI network. **(B)** Hub genes of **(A)** annua for the treatment of AAA.

The 117 targets of *A. annua* for the treatment of AAA were entered into the STRING platform; the parameter was set to *Homo sapiens*, with the highest confidence (0.900), and the rest of the parameters were set to default to build the target protein interaction network, as shown in Figure 6A. It was visualized and analysed by Cytoscape. A node in the PPI network represented each target, and the edges connecting the nodes represented the interaction between the targets. Topological analysis of 117 targets using the plug-in CytoNCA with two median filtering, the first filtering criterion was: betweenness: 31.368076265, closeness: 0.117252968, degree: 6, eigenvector: 0.0489839665, lac: 2.45, network: 3.3304473305. The second screening criterion was: betweenness: 8.080020796, closeness: 0.576923077, degree: 9. eigenvector: 0.145350322, lac: 4.5 network: 5.6, 11 hub genes were screened as shown in Figure 6B. RELA, APK14, CCND1, MAPK1, AKT1, MYC, MAPK8, TP53, ESR1, FOS, and JUN, with 11 nodes and 45 edges, scored as shown in Table 2. Therefore, we considered these 11 genes can serve as potential central genes of *A. annua* in the treatment of AAA.

**TABLE 2:** Hub genes of degree value in the PPI network.

name	Betweenness	Closeness	Degree	Eigenvector	LAC	Network
RELA	1.507,142,857	0.769,230,769	7	0.25,598,225	4.571,428,571	5.333,333,333
MYC	2.078,571,429	0.909,090,909	9	0.326,317,787	6.444,444,444	8.172,619,048
MAPK8	0.821,428,571	0.769,230,769	7	0.263,347,566	5.142,857,143	6
FOS	2.078,571,429	0.909,090,909	9	0.326,427,132	6.444,444,444	8.172,619,048
TP53	2.192,857,143	0.909,090,909	9	0.326,792,687	6.444,444,444	8.130,952,381
JUN	3.164,285,714	1	10	0.354,707,539	7	10
MAPK14	1.942,857,143	0.833,333,333	8	0.295,108,497	5.5	6.595,238,095
CCND1	1.257,142,857	0.769,230,769	7	0.263,187,557	4.857,142,857	5.666,666,667
ESR1	2.192,857,143	0.909,090,909	9	0.326,792,687	6.444,444,444	8.130,952,381
MAPK1	1.942,857,143	0.833,333,333	8	0.295,108,497	5.5	6.595,238,095
AKT1	0.821,428,571	0.769,230,769	7	0.26,312,241	5.142,857,143	6

### Molecular docking verification

**FIGURE 7:**
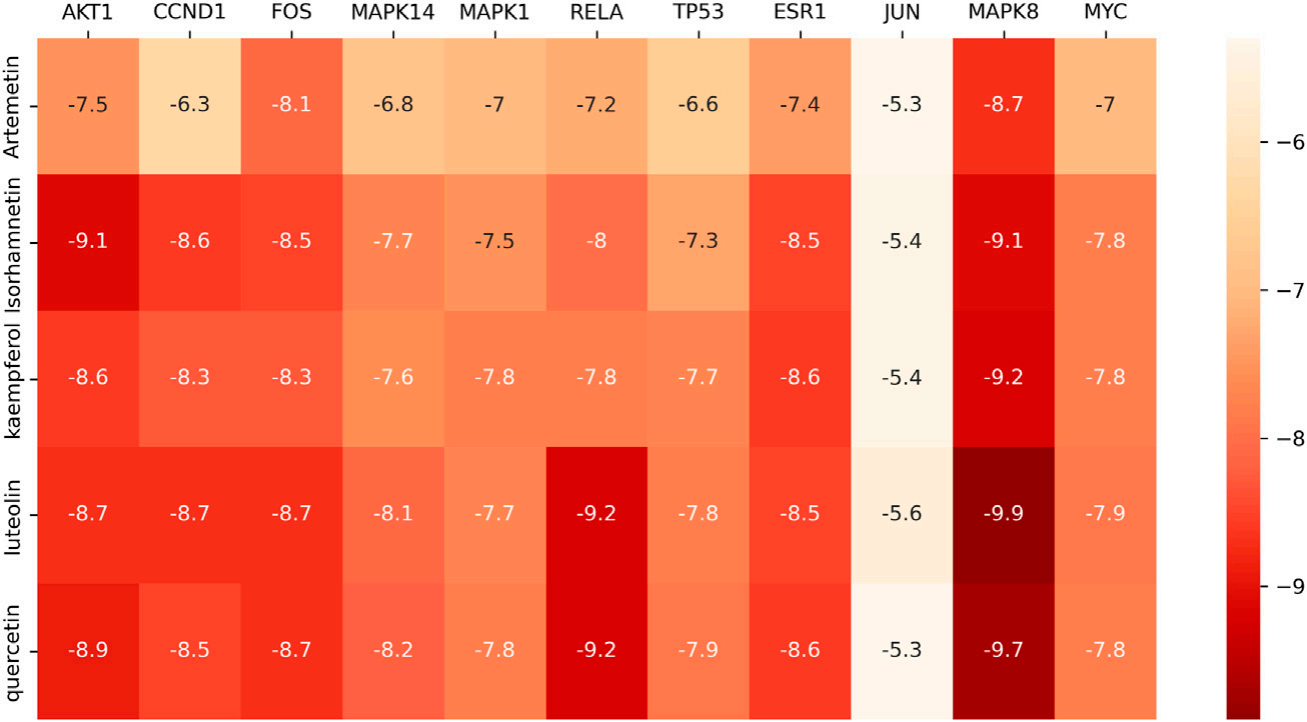
Thermal diagram of the molecular docking binding energy. The color from white to red indicated that the binding ability was weak to strong.

**FIGURE 8:**
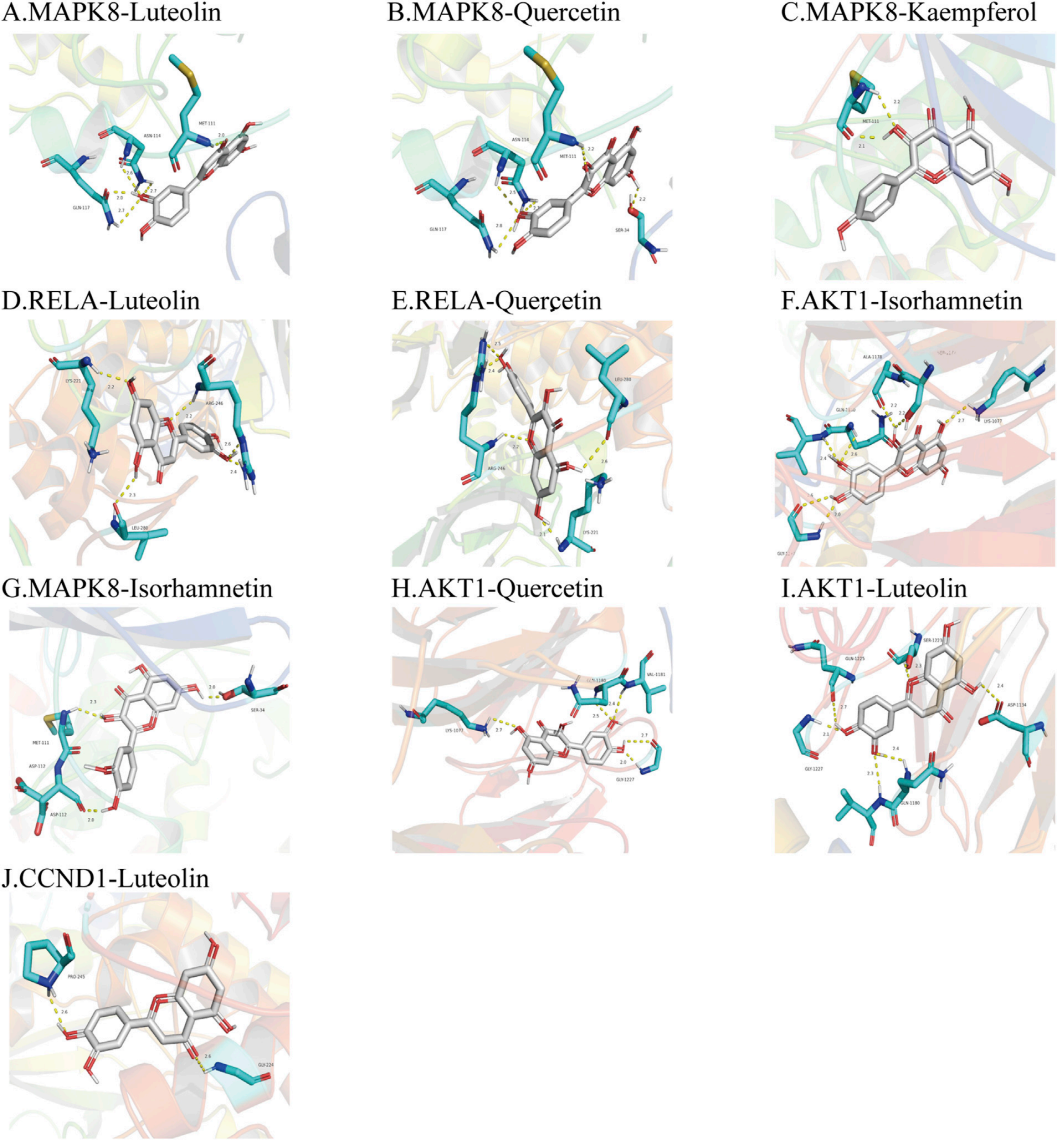
Molecular docking diagrams of the top 10 molecular docking maps with low binding energies. The protein active site, binding distance, and molecular docking model between the protein and the main active ingredient were shown in Figure 8. **(A)**. MAPK8-Luteolin (−9.9 kcal/mol); **(B)**. MAPK8-Quercetin (−9.7 kcal/mol); **(C)**. MAPK8-Kaempferol (−9.2 kcal/mol); **(D)**. RELA-Luteolin (−9.2 kcal/mol); **(E)**. RELA-Quercetin (−9.2 kcal/mol); **(F)**. AKT1-Isorhamnetin (−9.1 kcal/mol); **(G)**. MAPK8-Isorhamnetin (−9.1 kcal/mol); **(H)**. AKT1-Quercetin (−8.9 kcal/mol); **(I)**. AKT1-Luteolin (−8.7 kcal/mol); **(J)**. CCND1-Luteolin (−8.7 kcal/mol).

According to the “component-target” network, quercetin, luteolin, kaempferol, isorhamnetin, and artemetin were the top five active ingredients of *A. annua* in the treatment of AAA, 11 centers for topological analysis of genetic screening for potential *A. annua* center for gene therapy AAA. Therefore, we docked the active ingredients to the hub target genes. We downloaded the 3D structures of the five active ingredients from PubChem and the protein structures of eleven hub genes from the PDB database. All active ingredients and hub genes were docked, with binding free energies calculated by running Vina, and the results were presented as a thermal diagram in Figure 7. The binding free energy of less than or equal to −5.0 kcal/mol was regarded as good binding activity between molecules, and the binding free energy of less than or equal to −7 kcal/mol represented a strong binding force between molecules (Morris et al., 2008). The docking results showed that all five active ingredients had good affinity with eleven core disease targets and played a central role in the action of *A. annua* in the treatment of AAA. We showed the top 10 molecular docking maps with low binding energies in Figure 8. The low binding energy molecular docking results provided valuable information for the development of drugs to treat AAA.

## Discussion

Since the 2015 Nobel Prize in Physiology or Medicine was awarded to Chinese scientists, *Artemisia* has gained global attention (Efferth et al., 2015). *Artemisia* and its derivatives are used to treat malaria and are widely used to treat various oncological and cardiovascular diseases (Ahmad et al., 2015; Efferth, 2017; Lang et al., 2019; Feng et al., 2020; Jiang et al., 2020; Yin et al., 2020; Meng et al., 2021). As the COVID-19 epidemic rages around the world, some studies have been conducted to fully elucidate the mechanisms behind *A. annua*'s treatment of COVID-19 through network pharmacology and molecular docking techniques (Tang et al., 2022). More studies have been performed to explore the potential mechanisms of *A. annua* and the treatment of chronic hepatitis B and hepatocellular carcinoma (He et al., 2021; Zhang et al., 2021). With the development of network pharmacology, traditional medicine based on multi-component, multi-target, and multi-channel treatment of diseases have been paid more attention (Yuan et al., 2017).

Our research screened 22 potentially active ingredients of *A. annua*, including quercetin, luteolin, kaempferol, isorhamnetin, artemetin, and artemisinin. Some ingredients had been proven to affect vascular diseases, such as AAA. Quercetin, a flavonoid with anti-inflammatory activity, was the most abundant ingredients that could act on AAA and inhibited the development of AAA in mice (Wang et al., 2012). Additionally, quercetin attenuated neovascularization during AAA growth (Wang et al., 2020) and decreased oxidative stress in AAA mouse models (WANG et al., 2014). Luteolin inhibited vascular smooth muscle cells proliferation and migration (Xu et al., 2015; Wu et al., 2018). Kaempferol inhibited diabetic cardiomyopathy in rats through a hypoglycemic effect and upregulation of SIRT1 (Alshehri et al., 2021). Kaempferol attenuated atherosclerosis *via* the PI3K/AKT/Nrf2 pathway (Feng et al., 2021). In addition, some epidemiological studies had found a positive correlation between the consumption of foods containing kaempferol and a reduced risk of many diseases, such as cancer and cardiovascular disease (Calderón-Montaño et al., 2011). Isorhamnetin inhibited oxidative stress (Skalski et al., 2019), and prevented doxorubicin-induced cardiotoxicity (Sun et al., 2013), and isorhamnetin attenuated atherosclerosis through PI3K/AKT activation and HO-1-induced inhibition of macrophage apoptosis (Luo et al., 2015). *Artemetin* had a certain inhibitory effect on atherosclerosis (Kim and Shim, 2019) and also could reduce hypertension proliferation, migration, and inflammation of VSMCs (de Souza et al., 2011; Cao et al., 2015). Therefore, the above results also showed that the effective chemical components in *A. annua* had a certain therapeutic effect on treating AAA.

After our filtering, there are 22 active ingredients in Artemisia annua. The top compound is quercetin with a degree-value of 89, and the last compound with a degree-value of 1. The difference between the two values is too large. We predicted the compounds and targets that play a major role in *A. annua*, and the compounds with high degree-value have higher representativeness while referring to previous studies(Feng et al., 2022; Hong et al., 2022; Xu et al., 2022), we selected the top five compounds for subsequent validation and molecular docking. By screening a total of 117 intersections of a drug-disease gene, the topological analysis of 11 hub genes RELA, APK14, CCND1, MAPK1, AKT1, MYC, MAPK8, TP53, ESR1, FOS, and JUN were finally performed. The molecular docking technique predicted the binding strength between herbal components and targets (Zeng et al., 2019). The results showed that the top 5 active ingredients had a good affinity with 11 core disease targets, and the docking results were less than −5.0 kcal/mol, which further demonstrated that active ingredients and hub genes were critical targets of *A. annua* in the treating of AAA. Among these, MAPK8-Luteolin (−9.9 kcal/mol) had the best binding ability and could be used as a potential drug therapeutic target in the future. More literature had reported that these selected genes were closely related to AAA (DiMusto et al., 2012; Leeper et al., 2013; Ijaz et al., 2017; Hao et al., 2018; Zhang et al., 2018; Zhao et al., 2019; Moran et al., 2020). These genes and their associated pathways might become potential therapeutic targets for AAA treatment. Because there is no reliable high-level clinical evidence of drugs for the treatment of abdominal aortic aneurysms (Golledge, 2019), we do not have a positive control set in our molecular docking. As a result, based on the advantages of *A. annua* in multigene targeting, it is more promising to bring good news to AAA patients.

In addition, KEGG enrichment analysis showed that several pathways, such as the PI3K-Akt signaling pathway, fluid shear stress, atherosclerosis, and the AGE-RAGE signaling pathway, were closely associated with the treatment of AAA by *A. annua*.

The relationship between the above pathways and AAA had been extensively studied. Inhibition of Notch1-mediated inflammation prevented AAA *via* the PI3K/Akt signaling pathway (Ni et al., 2021), and AGE-RAGE stress was associated with the pathogenesis of aortic aneurysms (Prasad, 2019). Daidzein attenuated AAA through the NF-κB, p38-MAPK, and TGF-β1 pathways (Liu et al., 2016). The MAPK (mitogen-activated protein kinase)/ERK pathway was an essential regulator of AAA formation during matrix metalloproteinase (MMP) (Ghosh et al., 2012). Lithium chloride could inhibit AAA by modulating the NF-κB signaling pathway (Xu et al., 2021). Therefore, it was speculated that *A. annua* might inhibit AAA by acting on related signaling pathways or targets.

In summary, the study used network pharmacology and molecular docking technology strategies to predict the significant active compounds and critical targets of *A. annua* in the treatment of AAA and speculated the potential mechanisms from multiple approaches and perspectives. Therefore, *A. annua* might have multiple components, multiple targets, and multiple signaling pathways to play a role in treating AAA. Among them, quercetin corresponds to the most targets and has the strongest activity, and it will be one of the possible potential drugs in the future drug treatment of abdominal aortic aneurysm.

## Conclusion

Based on network pharmacology combined with molecular docking technology, this study systematically summarized the molecular targets of *A. annua* in the treatment of AAA, aiming to promote more comprehensive development and research of *A. annua*. The potential molecular mechanism of the active ingredients of *A. annua* in the treatment of AAA could support its subsequent clinical research and be vital for exploring the pharmacological treatment of AAA. At the same time, the current work also has some shortcomings. This study lacks corresponding experimental verification, which will be further verified in future research. In addition, the loss and incompleteness of some database information will also have a particular impact on the prediction results.

## Data Availability

The original contributions presented in the study are included in the article/Supplementary Material, further inquiries can be directed to the corresponding author.

## Abbreviations

AAA, abdominal aortic aneurysm; *A. annua*, *Artemisia annua* L; BP, biological process; CC, cellular component; DL, drug-likeness; GO, gene Ontology; KEGG, Kyoto encyclopedia of genes and Genomes; MF, molecular function; MMP, matrix metalloproteinase; OB, oral bioavailability; OMIM, online Mendelian inheritance in man; PDB, protein data bank; Pharm GKB, pharmacogenomics knowledgebase; PPI, protein-protein interaction; TCMSP, traditional Chinese medicines for systems pharmacology database and analysis platform; TTD, therapeutic target database.
